# Evaluation of the identification performance of MALDI-TOF MS Smart MS 5020 from Zhuhai DL Biotech

**DOI:** 10.1128/spectrum.00664-25

**Published:** 2025-06-12

**Authors:** Blanca Carrasco, Elena Hidalgo, Ángela Iniesta, Juan-Ignacio Alós

**Affiliations:** 1 Servicio de Microbiología, Hospital Universitario de Getafe 16503, Getafe, Madrid, Spain; The University of Arizona, Tucson, Arizona, USA

**Keywords:** MALDI-TOF, mass spectrometry, identification, Smart MS 5020, Biotyper Microflex LT

## Abstract

Matrix-assisted laser desorption/ionization-time of flight mass spectrometry (MALDI-TOF MS) is a reliable technique, is rapid and precise in the identification of pathogens, and is essential in clinical microbiology laboratories. The aim of this project is to evaluate the identification performance of the MALDI-TOF MS Smart MS 5020 system (Zhuhai DL Biotech) by comparing the identification results with those of the MALDI-TOF MS Biotyper Microflex LT system (Bruker Daltonics). A total of 612 clinical isolates were analyzed in parallel: 494 routine species and 118 species not commonly isolated in routine practice, previously isolated and frozen in our hospital’s strain collection. This collection included gram-negative bacteria, gram-positive bacteria, anaerobes, and fungi. Concordance at genus and species level identification was evaluated, and 16S rRNA gene sequencing was performed for strains unidentified or with discordant results. Both systems, Smart MS 5020 and Biotyper Microflex LT, achieved high identification rates (100% and 98.9%, respectively). The concordance between the systems was 98.9% at the genus level and 97.2% at the species level. Correct species-level identification was 96.9% for the Smart MS 5020 and 96.6% for the Biotyper Microflex LT. The MALDI-TOF MS Smart MS 5020 and Biotyper Microflex LT systems offer high efficacy in identifying a wide variety of clinical isolates, both demonstrating excellent performance.

**IMPORTANCE:**

Accurate identification of microorganisms in clinical microbiology laboratories is essential for the effective diagnosis and treatment of patients. This study evaluates the performance of the Smart MS 5020 matrix-assisted laser desorption/ionization-time-of-flight mass spectrometry (MALDI-TOF MS) system in comparison to the widely used Biotyper Microflex LT system. As a new entrant to the market, independent validation of the Smart MS 5020 system is crucial for laboratories considering its implementation. By analyzing 612 clinical isolates, including both routine and rare species, this study provides valuable data on the concordance rates between the two systems. The results confirm the Smart MS 5020 high accuracy and reinforce the reliability of MALDI-TOF MS technology. The high concordance rate between the two systems establishes the Smart MS 5020 as a promising, cost-effective alternative for routine diagnostics. These results expand the evidence base on MALDI-TOF MS platforms and can inform procurement decisions in clinical laboratories worldwide.

## INTRODUCTION

In the microbiological diagnosis of infections, rapid and accurate pathogen identification by clinical microbiology laboratories is crucial for ensuring favorable patient outcomes (1). Identification of cultured pathogens by matrix-assisted laser desorption/ionization-time-of-flight mass spectrometry (MALDI-TOF MS) is reliable, timely, and cost-effective (2, 3). Consequently, it has become a standard tool in clinical microbiology laboratories in developed healthcare systems.

Currently, two manufacturers dominate the global market: Bruker Daltonics and bioMérieux (4, 5). In 2019, a new competitor from China, Zhuhai DL Biotech Co., Ltd, entered the market. However, the availability of this MALDI-TOF MS system has been largely limited to local markets, and performance data for the device are scarce. Only one study, published in Chinese and not indexed in PubMed, has evaluated its performance (6).

The objective of this study was to evaluate the identification performance of the MALDI-TOF MS Smart MS 5020 (Zhuhai DL Biotech, P.R. China) by comparing its results with those obtained using the MALDI-TOF MS Biotyper Microflex LT (Bruker Daltonics, Germany). Identification rates and concordance between the two systems were assessed.

## MATERIALS AND METHODS

This study included 612 clinical isolates from the Hospital Universitario de Getafe (Spain), comprising 264 gram-negative bacteria, 240 gram-positive bacteria, 52 anaerobic bacteria, and 56 fungi (Table 1). Isolates were prospectively collected from routine laboratory samples (*n* = 494) and frozen preserved strains from laboratory collection (*n* = 118), representing the diversity of microorganisms typically found in clinical microbiology laboratories.

**TABLE 1:** Clinical isolates of the study at the genus level

All isolates	Genus	*N*	%
Enterobacterales			
	*Citrobacter*	9	1.4
	*Enterobacter*	25	4.1
	*Escherichia*	27	4.4
	*Klebsiella*	34	5.6
	*Morganella*	4	0.6
	*Proteus*	17	2.8
	*Pantoea*	1	0.2
	*Raoultella*	4	0.6
	*Rahnella*	2	0.3
	*Serratia*	10	1.6
	*Yersinia*	1	0.2
	*Hafnia*	2	0.3
	*Plesiomonas*	2	0.3
	*Cronobacter*	1	0.2
	*Salmonella*	7	1.1
	*Leclercia*	1	0.2
	*Cedecea*	1	0.2
Non-fermenting gram-negative bacilli			
	*Achromobacter*	7	1.1
	*Acinetobacter*	18	2.9
	*Burkholderia*	6	0.9
	*Comamonas*	2	0.3
	*Pseudomonas*	28	4.6
	*Elizabethkingia*	2	0.3
	*Stenotrophomonas*	6	0.9
	*Sphingobacterium*	1	0.2
	*Roseomonas*	1	0.2
	*Chryseobacterium*	1	0.2
	*Delftia*	1	0.2
	*Flavobacterium*	1	0.2
	*Pandoraea*	2	0.3
Other Gram-negative			
	*Campylobacter*	9	1.4
	*Aeromonas*	6	0.9
	*Aggregatibacter*	2	0.3
	*Vibrio*	1	0.2
	*Pasteurella*	1	0.2
	*Haemophilus*	4	0.6
	*Kingella*	2	0.3
	*Neisseria*	8	1.3
	*Moraxella*	6	0.9
	*Eikenella*	1	0.2
Gram-positive cocci			
	*Staphylococcus*	73	11.9
	*Macrococcus*	1	0.2
	*Enterococcus*	32	5.2
	*Streptococcus*	56	9.1
	*Kocuria*	2	0.3
	*Globicatella*	1	0.2
	*Aerococcus*	1	0.2
	*Leuconostoc*	3	0.5
	*Atopobium*	1	0.2
	*Helcococcus*	1	0.2
	*Lactococcus*	1	0.2
	*Micrococcus*	1	0.2
	*Dermacoccus*	1	0.2
	*Gemella*	1	0.2
Gram-positive rods			
	*Listeria*	3	0.5
	*Priestia*	1	0.2
	*Corynebacterium*	20	3.3
	*Lactobacillus*	12	1.9
	*Dermabacter*	4	0.6
	*Pseudoglutamicibacter*	3	0.5
	*Turicella*	2	0.3
	*Actinotignum*	1	0.2
	*Paenibacillus*	1	0.2
	*Nocardia*	2	0.3
	*Bacillus*	6	0.9
	*Lysinibacillus*	2	0.3
	*Trusperella*	1	0.2
	*Arcanobacterium*	1	0.2
	*Alloscardovia*	1	0.2
	*Weissella*	1	0.2
Anaerobes			
	*Peptostreptococcus*	1	0.2
	*Finegoldia*	3	0.5
	*Propionibacterium*	1	0.2
	*Bacteroides*	16	2.6
	*Parabacteroides*	1	0.2
	*Cutibacterium*	4	0.6
	*Clostridium*	7	1.1
	*Actinomyces*	4	0.6
	*Enterocloster*	1	0.2
	*Fusobacterium*	3	0.5
	*Prevotella*	4	0.6
	*Peptoniphilus*	3	0.5
	*Parvimonas*	1	0.2
	*Bilophila*	1	0.2
	*Veillonella*	1	0.2
	*Alistipes*	1	0.2
	*Lactococcus*	1	0.2
	*Eubacterium*	1	0.2
	*Anaerococcus*	2	0.3
Fungi			
	*Candida*	40	6.5
	*Kluyveromyces*	1	0.2
	*Aspergillus*	10	1.6
	*Fusarium*	1	0.2
	*Saccharomyces*	1	0.2
	*Scedosporium*	1	0.2
	*Trichophyton*	2	0.3

Six American Type Culture Collection (ATCC) strains were included to ensure reproducibility: ATCC 8739 and ATCC 35218 (*Escherichia coli*), ATCC 29213 (*Staphylococcus aureus*), ATCC 27853 (*Pseudomonas aeruginosa*), ATCC 700603 (*Klebsiella pneumoniae*), and ATCC 29212 (*Enterococcus faecalis*). These strains were tested after every 150 isolates processed by each MALDI-TOF MS system.

An *Escherichia coli* ATCC 8739 strain was used to calibrate the Smart MS 5020 instrument according to the manufacturer’s recommendations. Neither positive nor negative controls are required for the operation of the system.

All isolates were identified on a colony basis.

For the study, MALDI-TOF MS Smart MS 5020 (Zhuhai DL Biotech, P.R. China) and Biotyper Microflex LT (Bruker Daltonics, Bremen, Germany) systems were compared in parallel.

### Isolate preparation

Prior to the identification of fresh colonies, all strains were subcultured; aerobic bacteria were grown on blood agar or chocolate agar plates (bioMérieux, Marcy-l'Étoile, France) and anaerobic bacteria on Schaedler agar (bioMérieux, Marcy-l'Étoile, France). Plates were incubated at 37°C for 18 ± 2 hours. Yeasts and filamentous fungi were grown on Sabouraud agar (bioMérieux, Marcy-l'Étoile, France) and incubated at 37°C or 30°C as needed.

### MALDI-TOF MS systems

#### Smart MS 5020

The Smart MS 5020 system (Zhuhai DL Biotech) uses DL Mass software with a database containing 2,692 species (2023 version).

The instrument uses a reusable plate.

Sample preparation followed the manufacturer’s protocol for strain identification. For bacteria, the direct smear method with formic acid (1 µL) was used, while for yeasts and filamentous fungi, both formic acid (1 µL) and ethanol (1 µL) were used. The plate was placed in the instrument, and the spectra of each isolate were compared with those in the database.

#### Biotyper Microflex LT

The Biotyper Microflex LT system (Bruker Daltonics) operates with MBT Compass software. The current database is the MBT Compass Library Revision K, which contains a total of 4,274 species.

Sample preparation followed the manufacturer’s protocol: the direct smear method for bacteria and formic acid (1 µL) treatment for yeasts and fungi. The plate was inserted into the instrument, and the spectrum of each isolate was compared with the spectra in the database.

The instrument uses a reusable plate.

### Identification criteria

Identification in both MALDI-TOF MS systems was given by a score indicating the confidence level of the identification results for each sample.

MALDI-TOF MS Biotyper Microflex LT identification scores are generally interpreted as follows:

- 2.0–3.0: High confidence identification.
- 1.70–1.99: Low confidence identification.
- 0.00–1.69: No identification of organisms possible.

MALDI-TOF Smart MS 5020 identification scores are generally interpreted as shown:

- ≥2.0: Identification certain to species level.
- 1.7–1.9: Identification certain to genus level and probable to species level.
- <1.7: Unsure identification, further review may be necessary.

If identification to species level was not achieved, a new subculture was performed, and the identification was repeated using both systems.

The rate of identification of each instrument was the percentage of strains with at least a probable identification at the species level.

### Comparison of the identification results

Concordance between systems was assessed at the genus and species levels. A correct identification was considered when the identification results of both systems were the same and with a reliable score of >1.7.

16S rRNA gene sequencing was carried out in cases where identifications generated by both MALDI-TOF MS systems yielded discordant or inconclusive results (7). Taxonomic classification at the species level was achieved through full-length sequencing of the 16S rRNA gene (~1,215 bp), with subsequent proofreading and sequence curation as required. The resulting sequences were compared against entries in the GenBank database using the BLAST+ 2.16.0 algorithm (http://www.ncbi.nlm.nih.gov/BLAST) and those exhibiting ≥99.6% similarity were considered conspecific and thus positively identified, in accordance with CLSI MM18 guidelines (8).

### Statistical analysis

*χ*^2^ test was used to compare the different identification rates between both systems. Findings were considered significant if the *p* value was <0.05.

## RESULTS

Both systems achieved high identification rates: 100% for the Smart MS 5020 and 99.8% for the Biotyper Microflex LT. The identification results of both systems are shown in Table 2; Table S1 in Supplementary Material. Concordance between systems was 98.9% at the genus level and 97.2% at the species level.

**TABLE 2:** Identification results of clinical isolates

		Smart MS 5020			Biotyper Microflex LT		
Group of organisms	*N*	Species (%)	Genus (%)	No reliable ID (%)	Species (%)	Genus (%)	No reliable ID (%)
Enterobacterales	148	140 (94.6)	8 (5.4)	0 (0.0)	140 (94.6)	8 (5.4)	0 (0.0)
Nonfermenters	76	72 (94.7)	4 (5.3)	0 (0.0)	74 (97.4)	1 (1.3)	1 (1.3)
Other gram-negative	40	40 (100.0)	0 (0.0)	0 (0.0)	40 (100.0)	0 (0.0)	0 (0.0)
Gram-positive cocci	175	173 (98.9)	2 (1.1)	0 (0.0)	175 (100.0)	0 (0.0)	0 (0.0)
Gram-positive rods	61	58 (95.1)	3 (4.9)	0 (0.0)	58 (95.1)	3 (4.9)	0 (0.0)
Anaerobes	56	54 (96.4)	2 (3.6)	0 (0.0)	54 (96.4)	2 (3.6)	0 (0.0)
Fungi	56	56 (100.0)	0 (0.0)	0 (0.0)	50 (89.3)	0 (0.0)	6 (10.7)
Total	612	593 (96.9)	19 (3.1)	0 (0.0)	591 (96.6)	14 (2.3)	7 (1.1)

The sequencing results of the 16S rRNA gene of 10 strains—either unidentified or with discordant results between both systems—are shown in Table 3.

**TABLE 3:** 16S rRNA gene sequencing results of unidentified or discordant strains

16S rRNA	Smart MS 5020	Biotyper Microflex LT
*Acinetobacter bereziniae*	*Acinetobacter* sp.	*Acinetobacter bereziniae*
*Chryseobacterium* sp.	*Chryseobacterium indologenes*	No identification
*Pandoraea commovens*	*Pandoraea apista*	*Pandoraea sputorum*
*Cronobacter sakazakii*	*Cronobacter sakazakii*	*Cronobacter* sp.
*Peptoniphilus* sp.	*Peptoniphilus gorbachii*	*Peptoniphilus grossensis*
*Streptococcus canis*	*Streptococcus dysgalactiae*	*Streptococcus canis*
*Acinetobacter proteolyticus*	*Acinetobacter junii/haemolyticus*	*Acinetobacter proteolyticus*
*Bacillus horneckiae*	*Bacillus infantis/firmus*	*Cytobacillus horneckiae*
*Cedecea lapagei*	*Cedecea neteri*	*Cedecea lapagei*
*Leuconostoc mesenteroides*	*Leuconostoc* sp.	*Leuconostoc mesenteroides*

The correct identification of the species level using sequencing results was achieved in 96.9% of cases (593/612) with the MALDI-TOF Smart MS 5020 system, compared to 96.6% (591/612) with the MALDI-TOF Biotyper Microflex LT system (*χ*² =0.1034; *p* = 0.74).

### ATCC strains

After performing four parallel tests for each of the six ATCC control strains, all were correctly identified (100%) with an identification score >2.

### Bacterial isolates

The Smart MS 5020 system correctly identified 537 bacterial isolates (96.6%) at the species level and 19 (3.4%) at the genus level. In the case of the Biotyper Microflex LT system, 542 bacterial isolates (97.5%) were correctly identified at the species level, 11 (2.0%) only at the genus level, and in one case (0.2%), there was no reliable identification. Most strains that were identified only at the genus level corresponded to *Salmonella* in both systems.

The strain not identified by the Biotyper Microflex LT system corresponded to the genus *Chryseobacterium* (Table 3).

### Fungal isolates

The Smart MS 5020 system accurately identified all 56 fungal isolates (100%), whereas the Biotyper Microflex LT identified 89.3% of fungal isolates.

## DISCUSSION

MALDI-TOF mass spectrometry is a fundamental method for species-level identification of pathogenic microorganisms.

The MALDI-TOF MS Smart MS 5020 system is a registered system launched by Zhuhai DL in 2021 (6). The evaluation of this equipment’s identification performance has only been published in a paper in Chinese, which is not indexed in PubMed (6).

The present study provides a comparative analysis of the identification performance of two MALDI-TOF MS systems: the Smart MS 5020 (Zhuhai DL Biotech, China) and the Biotyper Microflex LT (Bruker Daltonics, Germany). The main objective was to evaluate the performance of the Smart MS 5020 system in identifying a wide range of clinical isolates, including both routine and unusual bacterial and fungal species.

When comparing our results with those obtained in the study conducted in China in 2021, the species-level identification performance of the Smart MS 5020 system was lower, at 96.6% compared to 99.78% in the previous study. We attribute this difference to the large number of species included in our study, many of which are uncommon in daily clinical laboratory practice.

The results indicate that both systems demonstrate high identification performance, with species-level identification rates of 96.9% for the Smart MS 5020 and 96.6% for the Biotyper Microflex LT. These findings suggest that both systems are highly effective in identifying clinical isolates. The results of the *χ*^2^ test indicate that there is no statistically significant difference between the two systems, supporting the conclusion that both systems perform effectively in the clinical setting.

The use of reference microorganisms (ATCC strains) for the validation and comparative analysis of both MALDI-TOF systems confirms the reproducibility of both systems for routine use in clinical microbiology.

In terms of fungal identification, the Smart MS 5020 accurately identified all 56 fungal isolates included in the study. In comparison, the Biotyper Microflex LT system correctly identified 89.3% of the fungal isolates, with notable difficulties in accurately identifying *Aspergillus fumigatus*.

One of the possible limitations of our study is the small variety of fungal species included. This is due to the fact that most clinical isolates in our hospital are of *Aspergillus* and *Candida* species.

Although both systems demonstrated high performance, some bacteria, such as *Salmonella* spp., were only identified at the genus level, reducing the accuracy of species identification. This limitation is a known drawback of MALDI-TOF MS systems due to the diversity and complexity within this genus (9).

An important aspect of our study is the inclusion of a large number of diverse strains in terms of genus and species, encompassing both species commonly encountered in daily laboratory routines and those rarely isolated, allowing for a more detailed evaluation of the identification capabilities of the equipment. These findings suggest that both the Smart MS 5020 and Biotyper Microflex LT are highly reliable devices for identifying a wide range of clinical isolates.

### Conclusion

The MALDI-TOF MS Smart MS 5020 and Biotyper Microflex LT systems demonstrate high accuracy and reliability in identifying clinical isolates. Continuous updates of software databases in both systems will further enhance their performance and utility in clinical microbiology.

## AFTER EPUB

[This article was published on 12 June 2025 with errors in Author Contribution and a misplacement of Funding statement. The errors were corrected in the current version, posted on 25 June 2025.]

## SUPPLEMENTAL MATERIAL

Table S1 spectrum.00664-25-s0001.docx Comparison of identification performance in clinical strains.

ASM does not own the copyrights to Supplemental Material that may be linked to, or accessed through, an article. The authors have granted ASM a non-exclusive, world-wide license to publish the Supplemental Material files. Please contact the corresponding author directly for reuse.

## References

[1] Vallicelli C, Santandrea G, Sartelli M, Coccolini F, Ansaloni L, Agnoletti V, Bravi F, Catena F. 2022. Sepsis team organizational model to decrease mortality for intra-abdominal infections: is antibiotic stewardship enough? Antibiotics (Basel) 11:1460. doi:10.3390/antibiotics11111460 36358115 PMC9687019

[2] Chen XF, Hou X, Xiao M, Zhang L, Cheng JW, Zhou ML, Huang JJ, Zhang JJ, Xu YC, Hsueh PR. 2021. Matrix-assisted laser desorption/ionization time of flight mass spectrometry (MALDI-TOF MS) analysis for the identification of pathogenic microorganisms: a review. Microorganisms 9:1536. doi:10.3390/microorganisms9071536 34361971 PMC8304613

[3] Oviaño M, Rodríguez-Sánchez B. 2021. MALDI-TOF mass spectrometry in the 21st century clinical microbiology laboratory. Enferm Infecc Microbiol Clin (Engl Ed) 39:192–200. doi:10.1016/j.eimc.2020.02.027 32345492

[4] Thelen P, Graeber S, Schmidt E, Hamprecht A. 2023. A side-by-side comparison of the new VITEK MS PRIME and the MALDI Biotyper sirius in the clinical microbiology laboratory. Eur J Clin Microbiol Infect Dis 42:1355–1363. doi:10.1007/s10096-023-04666-x 37794128 PMC10587274

[5] Bilecen K, Yaman G, Ciftci U, Laleli YR. 2015. Performances and reliability of Bruker Microflex LT and VITEK MS MALDI-TOF mass spectrometry systems for the identification of clinical microorganisms. Biomed Res Int 2015:516410. doi:10.1155/2015/516410 26793718 PMC4697076

[6] Li J, Yonglu H, Jiaping L, Chenchen J, Hongwei Z, Rong Z, Yanyan H. 2022. Evaluation for performance of domestic MALDI-TOF MS Smart MS 5020 in identification of clinical microorganisms. Chin J Clin Lab Sci 40:8. doi:10.13602/j.cnki.jcls.2022.08.04

[7] Dichtl K, Klugherz I, Greimel H, Luxner J, Köberl J, Friedl S, Steinmetz I, Leitner E. 2023. A head-to-head comparison of three MALDI-TOF mass spectrometry systems with 16S rRNA gene sequencing. J Clin Microbiol 61:e0191322. doi:10.1128/jcm.01913-22 37732759 PMC10595064

[8] Petti CA, Bosshard PP, Brandt ME, Clarridge JE, Feldblyum TV, Foxall P, et al.. 2008. Interpretive criteria for identification of bacteria and fungi by DNA target sequencing: approved guidelines. Vol. 28. “Isolation and identification of kroppenstedtia eburnea isolates from ...”. Clinical and Laboratory Standards Institute, Wayne, PA.

[9] Chen SH, Parker CH, Croley TR, McFarland MA. 2021. Genus, species, and subspecies classification of salmonella isolates by proteomics. Appl Sci (Basel) 11:4264. doi:10.3390/app11094264

