# Supplementary material for: Evaluation of the identification performance of MALDI-TOF MS Smart MS 5020 from Zhuhai DL Biotech
Source: Microbiol Spectr. 2025 Jun 12;13(8):e00664-25. doi: 10.1128/spectrum.00664-25 (PMC12323580; doi:10.1128/spectrum.00664-25)
Supplement: Table S1 — Comparison of identification performance in clinical strains. [file spectrum.00664-25-s0001.docx]

**Supplementary Materials**

**Table 1S. Comparison of identification performance in clinical strains.**

|  |  | **Smart MS 5020** | | | | **Biotyper Microflex LT** | | | |
| --- | --- | --- | --- | --- | --- | --- | --- | --- | --- |
| **Species** | ***N*** | **ID species** | **ID genus** | **No ID** | **MisID** | **ID species** | **ID genus** | **No ID** | **MisID** |
| **Enterobacterales (%)** | 148 | 140 (94.6) | 8 (5.4) | 0 (0.0) | 0 (0.0) | 140 (94.6) | 8 (5.4) | 0 (0.0) | 0 (0.0) |
| *Citrobacter koseri* | 2 | 2 | 0 | 0 | 0 | 2 | 0 | 0 | 0 |
| *Citrobacter farmeri* | 1 | 1 | 0 | 0 | 0 | 1 | 0 | 0 | 0 |
| *Citrobacter freundii complex* | 5 | 5 | 0 | 0 | 0 | 5 | 0 | 0 | 0 |
| *Citrobacter amalonaticus** | 1 | 1 | 0 | 0 | 0 | 1 | 0 | 0 | 0 |
| *Enterobacter cloacae complex* | 25 | 25 | 0 | 0 | 0 | 25 | 0 | 0 | 0 |
| *Escherichia coli* | 26 | 26 | 0 | 0 | 0 | 26 | 0 | 0 | 0 |
| *Escherichia vulneris** | 1 | 1 | 0 | 0 | 0 | 1 | 0 | 0 | 0 |
| *Klebsiella aerogenes* | 1 | 1 | 0 | 0 | 0 | 1 | 0 | 0 | 0 |
| *Klebsiella oxytoca* | 9 | 9 | 0 | 0 | 0 | 9 | 0 | 0 | 0 |
| *Klebsiella pneumoniae complex* | 24 | 24 | 0 | 0 | 0 | 24 | 0 | 0 | 0 |
| *Morganella morganii* | 4 | 4 | 0 | 0 | 0 | 4 | 0 | 0 | 0 |
| *Proteus mirabilis* | 12 | 12 | 0 | 0 | 0 | 12 | 0 | 0 | 0 |
| *Proteus vulgaris* | 5 | 5 | 0 | 0 | 0 | 5 | 0 | 0 | 0 |
| *Pantoea antophila** | 1 | 1 | 0 | 0 | 0 | 1 | 0 | 0 | 0 |
| *Raoultella ornithinolytica* | 2 | 2 | 0 | 0 | 0 | 2 | 0 | 0 | 0 |
| *Raoultella planticola* | 2 | 2 | 0 | 0 | 0 | 2 | 0 | 0 | 0 |
| *Rahnella aquatilis** | 2 | 2 | 0 | 0 | 0 | 2 | 0 | 0 | 0 |
| *Serratia marcescens* | 7 | 7 | 0 | 0 | 0 | 7 | 0 | 0 | 0 |
| *Serratia liquefaciens* | 2 | 2 | 0 | 0 | 0 | 2 | 0 | 0 | 0 |
| *Serratia rubidaea** | 1 | 1 | 0 | 0 | 0 | 1 | 0 | 0 | 0 |
| *Yersinia enterocolitica** | 1 | 1 | 0 | 0 | 0 | 1 | 0 | 0 | 0 |
| *Hafnia alvei* | 2 | 2 | 0 | 0 | 0 | 2 | 0 | 0 | 0 |
| *Plesiomonas shigelloides* | 2 | 2 | 0 | 0 | 0 | 2 | 0 | 0 | 0 |
| *Cronobacter sakazakii** | 1 | 1 | 0 | 0 | 0 | 0 | 1 | 0 | 0 |
| *Salmonella specie* | 7 | 0 | 7 | 0 | 0 | 0 | 7 | 0 | 0 |
| *Leclercia adecarboxylata** | 1 | 1 | 0 | 0 | 0 | 1 | 0 | 0 | 0 |
| *Cedecea lapagei** | 1 | 0 | 1 | 0 | 0 | 1 | 0 | 0 | 0 |
| **Nonfermenters (%)** | 76 | 72 (94.7) | 4 (5.3) | 0 (0.0) | 0 (0.0) | 74 (97.4) | 1 (1.3) | 1 (1.3) | 0 (0.0) |
| *Achromobacter xylosoxidans* | 6 | 6 | 0 | 0 | 0 | 6 | 0 | 0 | 0 |
| *Achromobacter denitrificans** | 1 | 1 | 0 | 0 | 0 | 1 | 0 | 0 | 0 |
| *Acinetobacter baumannii complex* | 6 | 6 | 0 | 0 | 0 | 6 | 0 | 0 | 0 |
| *Acinetobacter ursingii* | 4 | 4 | 0 | 0 | 0 | 4 | 0 | 0 | 0 |
| *Acinetobacter proteolyticus** | 1 | 0 | 1 | 0 | 0 | 1 | 0 | 0 | 0 |
| *Acinetobacter towneri** | 1 | 1 | 0 | 0 | 0 | 1 | 0 | 0 | 0 |
| *Acinetobacter radioresistens* | 2 | 2 | 0 | 0 | 0 | 2 | 0 | 0 | 0 |
| *Acinetobacter lwoffii** | 3 | 3 | 0 | 0 | 0 | 3 | 0 | 0 | 0 |
| *Acinetobacter bereziniae** | 1 | 0 | 1 | 0 | 0 | 1 | 0 | 0 | 0 |
|  |  |  |  |  |  |  |  |  |  |
| *Burkholderia cepacia** | 5 | 5 | 0 | 0 | 0 | 5 | 0 | 0 | 0 |
| *Burkholderia gladioli** | 1 | 1 | 0 | 0 | 0 | 1 | 0 | 0 | 0 |
| *Comamonas kerstersii** | 2 | 2 | 0 | 0 | 0 | 2 | 0 | 0 | 0 |
| *Pseudomonas aeruginosa* | 22 | 22 | 0 | 0 | 0 | 22 | 0 | 0 | 0 |
| *Pseudomonas fulva** | 1 | 1 | 0 | 0 | 0 | 1 | 0 | 0 | 0 |
| *Pseudomonas stutzeri* | 3 | 3 | 0 | 0 | 0 | 3 | 0 | 0 | 0 |
| *Pseudomonas monteilii** | 1 | 1 | 0 | 0 | 0 | 1 | 0 | 0 | 0 |
| *Pseudomonas plecoglossicida** | 1 | 1 | 0 | 0 | 0 | 1 | 0 | 0 | 0 |
| *Elizabethkingia meningoseptica* | 2 | 2 | 0 | 0 | 0 | 2 | 0 | 0 | 0 |
| *Stenotrophomonas maltophilia* | 6 | 6 | 0 | 0 | 0 | 6 | 0 | 0 | 0 |
| *Sphingobacterium thalpophilum** | 1 | 1 | 0 | 0 | 0 | 1 | 0 | 0 | 0 |
| *Roseomonas mucosa** | 1 | 1 | 0 | 0 | 0 | 1 | 0 | 0 | 0 |
| *Chryseobacterium sp** | 1 | 0 | 1 | 0 | 0 | 0 | 0 | 1 | 0 |
| *Delftia acidovorans** | 1 | 1 | 0 | 0 | 0 | 1 | 0 | 0 | 0 |
| *Flavobacterium lindanitolerans* | 1 | 1 | 0 | 0 | 0 | 1 | 0 | 0 | 0 |
| *Pandoraea commovens** | 1 | 0 | 1 | 0 | 0 | 0 | 1 | 0 | 0 |
| *Pandoraea norimbergensis* | 1 | 1 | 0 | 0 | 0 | 1 | 0 | 0 | 0 |
| **Other Gram-negative (%)** | 40 | 40 (100.0) | 0 (0.0) | 0 (0.0) | 0 (0.0) | 40 (100.0) | 0 (0.0) | 0 (0.0) | 0 (0.0) |
| *Campylobacter jejuni* | 6 | 6 | 0 | 0 | 0 | 6 | 0 | 0 | 0 |
| *Campylobacter coli* | 2 | 2 | 0 | 0 | 0 | 2 | 0 | 0 | 0 |
| *Campylobacter upsaliensis** | 1 | 1 | 0 | 0 | 0 | 1 | 0 | 0 | 0 |
| *Aeromonas veronii* | 4 | 4 | 0 | 0 | 0 | 4 | 0 | 0 | 0 |
| *Aeromonas caviae** | 1 | 1 | 0 | 0 | 0 | 1 | 0 | 0 | 0 |
| *Aeromonas hydrophila** | 1 | 1 | 0 | 0 | 0 | 1 | 0 | 0 | 0 |
| *Aggregatibacter aphrophimus* | 1 | 1 | 0 | 0 | 0 | 1 | 0 | 0 | 0 |
| *Aggregatibacter segnis* | 1 | 1 | 0 | 0 | 0 | 1 | 0 | 0 | 0 |
| *Vibrio cholerae** | 1 | 1 | 0 | 0 | 0 | 1 | 0 | 0 | 0 |
| *Pasteurella canis** | 1 | 1 | 0 | 0 | 0 | 1 | 0 | 0 | 0 |
| *Haemophilus influenzae* | 4 | 4 | 0 | 0 | 0 | 4 | 0 | 0 | 0 |
| *Kingella kingae** | 1 | 1 | 0 | 0 | 0 | 1 | 0 | 0 | 0 |
| *Kingella denitrificans ** | 1 | 1 | 0 | 0 | 0 | 1 | 0 | 0 | 0 |
| *Neisseria meningitidis** | 1 | 1 | 0 | 0 | 0 | 1 | 0 | 0 | 0 |
| *Neisseria gonorrhoeae* | 3 | 3 | 0 | 0 | 0 | 3 | 0 | 0 | 0 |
| *Neisseria flavescens subflava group* | 3 | 3 | 0 | 0 | 0 | 3 | 0 | 0 | 0 |
| *Neisseria sicca group** | 1 | 1 | 0 | 0 | 0 | 1 | 0 | 0 | 0 |
| *Moraxella catarrhalis* | 4 | 4 | 0 | 0 | 0 | 4 | 0 | 0 | 0 |
| *Moraxella atlantae** | 1 | 1 | 0 | 0 | 0 | 1 | 0 | 0 | 0 |
| *Moraxella osloensis** | 1 | 1 | 0 | 0 | 0 | 1 | 0 | 0 | 0 |
| *Eikenella corrodens** | 1 | 1 | 0 | 0 | 0 | 1 | 0 | 0 | 0 |
| **Gram-positive cocci (%)** | 175 | 173 (98.9) | 2 (1.1) | 0 (0.0) | 0 (0.0) | 175 (100.0) | 0 (0.0) | 0 (0.0) | 0 (0.0) |
| *Staphylococcus aureus* | 18 | 18 | 0 | 0 | 0 | 18 | 0 | 0 | 0 |
| *Staphylococcus epidermidis* | 21 | 21 | 0 | 0 | 0 | 21 | 0 | 0 | 0 |
| *Staphylococcus lugdundensis* | 5 | 5 | 0 | 0 | 0 | 5 | 0 | 0 | 0 |
| *Staphylococcus saprophyticus* | 4 | 4 | 0 | 0 | 0 | 4 | 0 | 0 | 0 |
| *Staphylococcus haemolyticus* | 6 | 6 | 0 | 0 | 0 | 6 | 0 | 0 | 0 |
| *Staphylococcus hominis* | 7 | 7 | 0 | 0 | 0 | 7 | 0 | 0 | 0 |
| *Staphylococcus simulans* | 2 | 2 | 0 | 0 | 0 | 2 | 0 | 0 | 0 |
| *Staphylococcus caprae* | 1 | 1 | 0 | 0 | 0 | 1 | 0 | 0 | 0 |
| *Staphylococcus warneri** | 1 | 1 | 0 | 0 | 0 | 1 | 0 | 0 | 0 |
| *Staphylococcus pasteuri** | 3 | 3 | 0 | 0 | 0 | 3 | 0 | 0 | 0 |
| *Staphylococcus pettenkoferi* | 3 | 3 | 0 | 0 | 0 | 3 | 0 | 0 | 0 |
| *Staphylococcus capitis* | 2 | 2 | 0 | 0 | 0 | 2 | 0 | 0 | 0 |
| *Macrococcus caseolyticus** | 1 | 1 | 0 | 0 | 0 | 1 | 0 | 0 | 0 |
| *Enterococcus faecalis* | 18 | 18 | 0 | 0 | 0 | 18 | 0 | 0 | 0 |
| *Enterococcus faecium* | 11 | 11 | 0 | 0 | 0 | 11 | 0 | 0 | 0 |
| *Enterococcus gallinarum* | 2 | 2 | 0 | 0 | 0 | 2 | 0 | 0 | 0 |
| *Enterococcus avium* | 1 | 1 | 0 | 0 | 0 | 1 | 0 | 0 | 0 |
| *Streptococcus pneumoniae* | 6 | 6 | 0 | 0 | 0 | 6 | 0 | 0 | 0 |
| *Streptococcus agalactiae* | 11 | 11 | 0 | 0 | 0 | 11 | 0 | 0 | 0 |
| *Streptococcus anginosus* | 6 | 6 | 0 | 0 | 0 | 6 | 0 | 0 | 0 |
| *Streptococcus constellatus* | 3 | 3 | 0 | 0 | 0 | 3 | 0 | 0 | 0 |
| *Streptococcus pyogenes* | 6 | 6 | 0 | 0 | 0 | 6 | 0 | 0 | 0 |
| *Streptococcus parasanguinis* | 4 | 4 | 0 | 0 | 0 | 4 | 0 | 0 | 0 |
| *Streptococcus dysgalactiae* | 2 | 2 | 0 | 0 | 0 | 2 | 0 | 0 | 0 |
| *Streptococcus mitis/oralis* | 4 | 4 | 0 | 0 | 0 | 4 | 0 | 0 | 0 |
| *Streptococcus thermophilus** | 1 | 1 | 0 | 0 | 0 | 1 | 0 | 0 | 0 |
| *Streptococcus canis* | 1 | 0 | 1 | 0 | 0 | 1 | 0 | 0 | 0 |
| *Streptococcus gallolyticus* | 3 | 3 | 0 | 0 | 0 | 3 | 0 | 0 | 0 |
| *Streptococcus gordonii* | 2 | 2 | 0 | 0 | 0 | 2 | 0 | 0 | 0 |
| *Streptococcus mutans* | 1 | 1 | 0 | 0 | 0 | 1 | 0 | 0 | 0 |
| *Streptococcus pleomorphus** | 1 | 1 | 0 | 0 | 0 | 1 | 0 | 0 | 0 |
| *Streptococcus salivarius* | 2 | 2 | 0 | 0 | 0 | 2 | 0 | 0 | 0 |
| *Streptococcus lutetiensis* | 2 | 2 | 0 | 0 | 0 | 2 | 0 | 0 | 0 |
| *Streptococcus vestibularis* | 1 | 1 | 0 | 0 | 0 | 1 | 0 | 0 | 0 |
| *Kocuria rhizophila** | 2 | 2 | 0 | 0 | 0 | 2 | 0 | 0 | 0 |
| *Globicatella sulfidifaciens** | 1 | 1 | 0 | 0 | 0 | 1 | 0 | 0 | 0 |
| *Aerococcus urinae* | 1 | 1 | 0 | 0 | 0 | 1 | 0 | 0 | 0 |
| *Leuconostoc mesenteroides** | 3 | 2 | 1 | 0 | 0 | 3 | 0 | 0 | 0 |
| *Atopobium vaginae** | 1 | 1 | 0 | 0 | 0 | 1 | 0 | 0 | 0 |
| *Helcococcus kunzii** | 1 | 1 | 0 | 0 | 0 | 1 | 0 | 0 | 0 |
| *Lactococcus garvieae** | 1 | 1 | 0 | 0 | 0 | 1 | 0 | 0 | 0 |
| *Micrococcus luteus* | 1 | 1 | 0 | 0 | 0 | 1 | 0 | 0 | 0 |
| *Dermacoccus nishinomaen** | 1 | 1 | 0 | 0 | 0 | 1 | 0 | 0 | 0 |
| *Gemella morbillorum** | 1 | 1 | 0 | 0 | 0 | 1 | 0 | 0 | 0 |
| **Gram-positive rods (%)** | 61 | 58 (95.1) | 3 (4.9) | 0 (0.0) | 0 (0.0) | 58 (95.1) | 3 (4.9) | 0 (0.0) | 0 (0.0) |
| *Listeria monocytogenes** | 3 | 3 | 0 | 0 | 0 | 3 | 0 | 0 | 0 |
| *Priestia megaterium** | 1 | 1 | 0 | 0 | 0 | 1 | 0 | 0 | 0 |
| *Corynebacterium amycolatum* | 2 | 2 | 0 | 0 | 0 | 2 | 0 | 0 | 0 |
| *Corynebacterium striatum* | 7 | 7 | 0 | 0 | 0 | 7 | 0 | 0 | 0 |
| *Corynebacterium urealyticum* | 2 | 2 | 0 | 0 | 0 | 2 | 0 | 0 | 0 |
| *Corynebacterium pseudodiphteriticum** | 2 | 2 | 0 | 0 | 0 | 2 | 0 | 0 | 0 |
| *Corynebacterium tuberculostearicum** | 1 | 1 | 0 | 0 | 0 | 1 | 0 | 0 | 0 |
| *Corynebacterium jeikeium** | 1 | 1 | 0 | 0 | 0 | 1 | 0 | 0 | 0 |
| *Corynebacterium simulans* | 1 | 1 | 0 | 0 | 0 | 1 | 0 | 0 | 0 |
| *Corynebacterium aurimucosum* | 3 | 3 | 0 | 0 | 0 | 3 | 0 | 0 | 0 |
| *Corynebacterium mucifaciens** | 1 | 1 | 0 | 0 | 0 | 1 | 0 | 0 | 0 |
| *Lactobacillus gasseri* | 4 | 4 | 0 | 0 | 0 | 4 | 0 | 0 | 0 |
| *Lactobacillus sakei** | 1 | 1 | 0 | 0 | 0 | 1 | 0 | 0 | 0 |
| *Lactobacillus paracasei subsp. paracasei* | 4 | 4 | 0 | 0 | 0 | 4 | 0 | 0 | 0 |
| *Lactobacillus rhamnosus** | 1 | 1 | 0 | 0 | 0 | 1 | 0 | 0 | 0 |
| *Lactobacillus sp.* | 2 | 0 | 2 | 0 | 0 | 0 | 2 | 0 | 0 |
| *Dermabacter hominis** | 4 | 4 | 0 | 0 | 0 | 4 | 0 | 0 | 0 |
| *Pseudoglutamicibacter cumminsii** | 3 | 3 | 0 | 0 | 0 | 3 | 0 | 0 | 0 |
| *Turicella otitidis** | 2 | 2 | 0 | 0 | 0 | 2 | 0 | 0 | 0 |
| *Actinotignum schaalii* | 1 | 1 | 0 | 0 | 0 | 1 | 0 | 0 | 0 |
| *Paenibacillus glucanolyticus* | 1 | 1 | 0 | 0 | 0 | 1 | 0 | 0 | 0 |
| *Nocardia cyriacigeorgica** | 1 | 1 | 0 | 0 | 0 | 1 | 0 | 0 | 0 |
| *Nocardia carnea** | 1 | 1 | 0 | 0 | 0 | 1 | 0 | 0 | 0 |
| *Bacillus pumilus* | 2 | 2 | 0 | 0 | 0 | 2 | 0 | 0 | 0 |
| *Bacillus cereus* | 2 | 2 | 0 | 0 | 0 | 2 | 0 | 0 | 0 |
| *Bacillus muralis* | 1 | 1 | 0 | 0 | 0 | 1 | 0 | 0 | 0 |
| *Bacillus horneckiae** | 1 | 0 | 1 | 0 | 0 | 0 | 1 | 0 | 0 |
| *Lysinibacillus sphaericus** | 2 | 2 | 0 | 0 | 0 | 2 | 0 | 0 | 0 |
| *Trusperella bernardiae** | 1 | 1 | 0 | 0 | 0 | 1 | 0 | 0 | 0 |
| *Arcanobacterium haemolyticum** | 1 | 1 | 0 | 0 | 0 | 1 | 0 | 0 | 0 |
| *Alloscardovia omnicolens** | 1 | 1 | 0 | 0 | 0 | 1 | 0 | 0 | 0 |
| *Weissella confusa** | 1 | 1 | 0 | 0 | 0 | 1 | 0 | 0 | 0 |
| **Anaerobes (%)** | 56 | 54 (96.4) | 2 (3.6) | 0 (0.0) | 0 (0.0) | 54 (96.4) | 2 (3.6) | 0 (0.0) | 0 (0.0) |
| *Peptostreptococcus anaerobius* | 1 | 1 | 0 | 0 | 0 | 1 | 0 | 0 | 0 |
| *Finegoldia magna* | 3 | 3 | 0 | 0 | 0 | 3 | 0 | 0 | 0 |
| *Propionibacterium granulosum** | 1 | 1 | 0 | 0 | 0 | 1 | 0 | 0 | 0 |
| *Bacteroides thetaiotaomicron* | 4 | 4 | 0 | 0 | 0 | 4 | 0 | 0 | 0 |
| *Bacteroides vulgatus (Phocaeicola vulgatus)* | 2 | 2 | 0 | 0 | 0 | 2 | 0 | 0 | 0 |
| *Bacteroides fragilis* | 6 | 6 | 0 | 0 | 0 | 6 | 0 | 0 | 0 |
| *Bacteroides salyersiae** | 1 | 1 | 0 | 0 | 0 | 1 | 0 | 0 | 0 |
| *Bacteroides uniformis** | 1 | 1 | 0 | 0 | 0 | 1 | 0 | 0 | 0 |
| *Bacteroides caccae** | 1 | 1 | 0 | 0 | 0 | 1 | 0 | 0 | 0 |
| *Bacteroides stercoris** | 1 | 1 | 0 | 0 | 0 | 1 | 0 | 0 | 0 |
| *Parabacteroides distasonis** | 1 | 1 | 0 | 0 | 0 | 1 | 0 | 0 | 0 |
| *Cutibacterium acnes* | 4 | 4 | 0 | 0 | 0 | 4 | 0 | 0 | 0 |
| *Clostridium perfringens* | 2 | 2 | 0 | 0 | 0 | 2 | 0 | 0 | 0 |
| *Clostridium tertium** | 1 | 1 | 0 | 0 | 0 | 1 | 0 | 0 | 0 |
| *Clostridium ramosum** | 1 | 1 | 0 | 0 | 0 | 1 | 0 | 0 | 0 |
| *Clostridium septicum** | 2 | 2 | 0 | 0 | 0 | 1 | 0 | 0 | 0 |
| *Clostridium paraputrificum** | 1 | 1 | 0 | 0 | 0 | 1 | 0 | 0 | 0 |
| *Actinomyces naeslundii** | 1 | 1 | 0 | 0 | 0 | 1 | 0 | 0 | 0 |
| *Actinomyces turicensis* | 1 | 1 | 0 | 0 | 0 | 1 | 0 | 0 | 0 |
| *Actinomyces europaeus** | 2 | 2 | 0 | 0 | 0 | 2 | 0 | 0 | 0 |
| *Enterocloster clostridioformis** | 1 | 1 | 0 | 0 | 0 | 1 | 0 | 0 | 0 |
| *Fusobacterium nucleatum subsp. animalis** | 1 | 1 | 0 | 0 | 0 | 1 | 0 | 0 | 0 |
| *Fusobacterium necrophorum subsp. necrophorum** | 1 | 1 | 0 | 0 | 0 | 1 | 0 | 0 | 0 |
| *Fusobacterium gonidiaformans** | 1 | 1 | 0 | 0 | 0 | 1 | 0 | 0 | 0 |
| *Prevotella oris** | 1 | 1 | 0 | 0 | 0 | 1 | 0 | 0 | 0 |
| *Prevotella denticola* | 1 | 1 | 0 | 0 | 0 | 1 | 0 | 0 | 0 |
| *Prevotella disiens** | 1 | 1 | 0 | 0 | 0 | 1 | 0 | 0 | 0 |
| *Prevotella intermedia* | 1 | 1 | 0 | 0 | 0 | 1 | 0 | 0 | 0 |
| *Peptoniphilus harei ** | 2 | 2 | 0 | 0 | 0 | 2 | 0 | 0 | 0 |
| *Peptoniphilus sp.** | 1 | 0 | 1 | 0 | 0 | 0 | 1 | 0 | 0 |
| *Parvimonas micra* | 1 | 1 | 0 | 0 | 0 | 1 | 0 | 0 | 0 |
| *Bilophila sp.** | 1 | 0 | 1 | 0 | 0 | 0 | 1 | 0 | 0 |
| *Veillonella parvula** | 1 | 1 | 0 | 0 | 0 | 1 | 0 | 0 | 0 |
| *Alistipes onderdonkii** | 1 | 1 | 0 | 0 | 0 | 1 | 0 | 0 | 0 |
| *Lactococcus garvieae* | 1 | 1 | 0 | 0 | 0 | 1 | 0 | 0 | 0 |
| *Eubacterium limosum** | 1 | 1 | 0 | 0 | 0 | 1 | 0 | 0 | 0 |
| *Anaerococcus hydrogenalis** | 2 | 2 | 0 | 0 | 0 | 2 | 0 | 0 | 0 |
| **Fungi (%)** | 56 | 56 (100.0) | 0 (0.0) | 0 (0.0) | 0 (0.0) | 50 (89.3) | 0 (0.0) | 6 (10.7) | 0 (0.0) |
| *Candida glabrata* | 7 | 7 | 0 | 0 | 0 | 7 | 0 | 0 | 0 |
| *Candida albicans* | 12 | 12 | 0 | 0 | 0 | 12 | 0 | 0 | 0 |
| *Candida dublinensis* | 1 | 1 | 0 | 0 | 0 | 1 | 0 | 0 | 0 |
| *Candida krusei* | 1 | 1 | 0 | 0 | 0 | 1 | 0 | 0 | 0 |
| *Candida parapsilosis* | 7 | 7 | 0 | 0 | 0 | 7 | 0 | 0 | 0 |
| *Candida metapsilosis** | 3 | 3 | 0 | 0 | 0 | 3 | 0 | 0 | 0 |
| *Candida tropicalis* | 6 | 6 | 0 | 0 | 0 | 6 | 0 | 0 | 0 |
| *Candida orthopsilosis* | 1 | 1 | 0 | 0 | 0 | 1 | 0 | 0 | 0 |
| *Candida dubliniensis* | 1 | 1 | 0 | 0 | 0 | 1 | 0 | 0 | 0 |
| *Candida guilliermondii** | 1 | 1 | 0 | 0 | 0 | 1 | 0 | 0 | 0 |
| *Kluyveromyces marxianus** | 1 | 1 | 0 | 0 | 0 | 1 | 0 | 0 | 0 |
| *Aspergillus fumigatus* | 5 | 5 | 0 | 0 | 0 | 1 | 0 | 4 | 0 |
| *Aspergillus flavus* | 5 | 5 | 0 | 0 | 0 | 5 | 0 | 0 | 0 |
| *Fusarium oxysporum** | 1 | 1 | 0 | 0 | 0 | 1 | 0 | 0 | 0 |
| *Scedosporium prolificans* | 1 | 1 | 0 | 0 | 0 | 1 | 0 | 0 | 0 |
| *Saccharomyces paradoxus* | 1 | 1 | 0 | 0 | 0 | 1 | 0 | 0 | 0 |
| *Trichophyton rubrum* | 1 | 1 | 0 | 0 | 0 | 0 | 0 | 1 | 0 |
| *Trichophyton tonsurans* | 1 | 1 | 0 | 0 | 0 | 0 | 0 | 1 | 0 |
| **Total (%)** | 612 | 593 (96.9) | 19 (3.1) | 0 (0.0) | 0 (0.0) | 591 (96.6) | 14 (2.3) | 7 (1.1) | 0 (0.0) |

* Iced preserved strains. ID:Identification
